# Learning disability as a determinant of digital behavior in adolescents with ADHD: a cross-sectional study

**DOI:** 10.1007/s00787-025-02802-w

**Published:** 2025-06-27

**Authors:** Sukrucan Kocabas, Ibrahim Adak, Oguz Bilal Karakus, Zeynep Ece Toksoy

**Affiliations:** 1 Department of Child and Adolescent Psychiatry, Sultanbeyli State Hospital, No. 60, Pasakoy Street, Battalgazi, Sultanbeyli , Istanbul, 34935 Turkey; 2 Department of Child and Adolescent Psychiatry, University of Health Sciences, Erenkoy Mental and Neurological Diseases Training and Research Hospital, Istanbul, Turkey; 3 Department of Child and Adolescent Psychiatry, Trabzon Kanuni Training and Research Hospital, Trabzon, Turkey

**Keywords:** Child psychiatry, Attention deficit hyperactivity disorder, Learning disabilities, Technology addiction

## Abstract

Problematic technology use (PTU) has become an increasingly relevant concern among children and adolescents, warranting examination of risk factors that might influence usage patterns. Although Attention-Deficit/Hyperactivity Disorder (ADHD) is frequently studied in relation to technology use, little is known about the additional impact of comorbid Learning disorders (LD) within this population. This study examines the impact of LD on PTU among children and adolescents with ADHD. A total of 172 participants aged 8–17 and their parents were interviewed regarding screen time, types of devices used, and digital activity patterns. Half (*n* = 86) were diagnosed with ADHD + LD, and the other half (*n* = 86) with only ADHD. Youth completed the Young Internet Addiction Scale, Internet Gaming Disorder Scale, Social Media Addiction Scale and Smartphone Addiction Scale. Parents of the ADHD + LD group completed the Mathematics and Literacy Assessment Scale to evaluate LD symptom severity. No significant difference in screen time per day was found between groups (*p* > 0.05). The ADHD + LD group used fewer device types (t_(170)_ = 3.30, *p* = 0.004), showing lower preference for mobile phones (X^2^_(1)_ = 4.79, *p* = 0.038) and personal computers (X^2^_(1)_ = 7.07, *p* = 0.016). They spent less time texting (*p* = 0.026), watching movies (*p* = 0.026) and videos (*p* = 0.041), listening to music (*p* = 0.004) and educational activities (*p* < 0.001). Gaming participation rates were similar, but Real-Time Strategy (*p* = 0.004), Multiplayer Online Battle Arena (*p* = 0.002), and Puzzle & Platform (*p* = 0.004) games were less preferred by the ADHD + LD group. Weak positive correlations were found between the number of screen-based devices owned and screen time per day (*r* = 0.204, *p* = 0.007), and device ownership duration and screen time per day (*r* = 0.282, *p* < 0.001). Scale scores did not differ significantly between groups, and Mathematics and Literacy Assessment Scale scores showed no correlation with other scale scores or screen time per day (*p* > 0.05). Although overall screen time and PTU severity were similar between groups, youth with ADHD and comorbid LD engaged in a narrower range of screen-based activities. These findings suggest that LD may shape digital behavior not in terms of quantity, but through differences in how technology is used.

## Introduction

Integration of technologies into our daily lives has advanced rapidly over the past decade. Today, what was accepted as space-age technology in the early 2000s is now a part of our daily routines. As a result, the Internet and screen-centric device use have become essential parts of our lives, reshaping work habits, social interactions, and entertainment options.

As children are raised in such tech-oriented environments, Problematic Technology Use (PTU) has become one of the most common problems in child and adolescent psychiatry clinical practice [1]. Parents often complain about their children spending countless hours watching videos, playing online games, scrolling through their favourite social media applications, avoiding their daily responsibilities. The rapidly evolving nature of the internet, digital technology and social media ‘trends’, make our efforts to keep up almost impossible, rendering most research ‘outdated’ all the time. So far, the literature has many descriptions related to dysfunctional technology use, like ‘pathologic internet use’, ‘problematic internet use’, ‘compulsive computer-internet use’, ‘smartphone addiction’, ‘social media addiction’ [2–6]. Although they may seem related, clinical presentations associated with these terms may differ significantly. Currently, Internet Gaming Disorder (IGD) is the closest diagnosis in the DSM; however, its criteria specifically focus on gaming-related issues and do not encompass non-gaming problematic use. Despite having clinical similarities with substance use disorders and being widely accepted as a behavioral addiction, IGD remains listed under ‘Conditions for Further Study’ in the DSM for now [7]. Given the diversity of definitions in the literature, we preferred to use the term PTU as a broad, inclusive concept that encompasses various patterns of maladaptive digital engagement without being restricted to specific categories such as gaming, smartphone, or social media use [8].

Regarding comorbidity between PTU and neurodevelopmental disorders a significant amount of research can be found in the literature, especially on Attention-Deficit Hyperactivity Disorder (ADHD) and PTU [9–11]. Considering the core symptoms of ADHD, such as deficits in self-regulation and impulsivity, it is expected that children with ADHD have greater difficulties regulating their relationship with technology and are more prone to problematic use compared to their peers. They are also more likely to experience negative outcomes of PTU, including emotional and behavioral problems, which overall have a greater impact on their quality of life [12, 13].

Some recent studies also suggest that there may be a relationship between PTU and Learning Disorders (LD), with findings indicating that individuals with LD exhibit higher rates of PTU compared to typically developing peers [14, 15]. Given that children with LD frequently face academic challenges, it is plausible that such difficulties may foster avoidance behavior—a pattern that has been empirically demonstrated in this population [16]. In addition, these difficulties may lead children and adolescents to seek peer approval or self-actualization in alternative domains, such as digital environments, where they may feel less disadvantaged. Previous studies have also demonstrated that higher levels of PTU are associated with avoidant coping and coping inflexibility, suggesting that maladaptive coping styles may mediate the relationship between psychosocial challenges and PTU [17]. As children and adolescents with LD experience greater daily life struggles compared to their peers, they might be more prone to relying on such strategies. Beyond these psychosocial mechanisms, several studies have identified similar executive function deficits in individuals with LD and in those exhibiting PTU, particularly in working memory, inhibitory control, and cognitive flexibility [18, 19]. These parallels suggest that the two groups may share common neurocognitive profiles, although the directionality of this relationship remains to be clarified. Considering the high comorbidity rates between ADHD and LD, we believe that the relationship between LD and PTU should be further evaluated in detail, particularly to determine whether there is an independent interaction between them, or the previous findings might be related to the comorbid ADHD. Based on this framework, our hypotheses were:

- Youth diagnosed with ADHD and comorbid LD exhibit higher levels of PTU compared to those with ADHD without comorbid LD.
- There is a positive correlation between the severity of LD symptoms and PTU levels, with higher symptom severity being associated with increased PTU.
- Patterns of tech use differ between youth with ADHD based on the presence or absence of comorbid LD.

## Materials and methods

Our study was designed as a cross-sectional study, evaluating the patients attending a Child and Adolescent Psychiatry outpatient clinic at a tertiary mental health hospital in Istanbul, Turkey. The patients who participated in the study were recruited for six months after the Clinical Ethics Committee approval (Approval date November 10, 2023; approval number 72) of the same hospital, between November 2023 and May 2024. Sample size was determined as a minimum of 172 patients, based on an analysis of previous studies [14, 15], with an alpha error %5, a power of 90%, and an effect size of 0.45.

Inclusion criteria for the study group were having ADHD and LD diagnoses based on the DSM-5-TR criteria, age between 8 and 17, no other psychiatric, neurological or chronic medical diagnosis, being literate and ongoing methylphenidate treatment for at least 6 months. The same inclusion criteria applied to the ADHD-only group, except for the absence of a LD diagnosis. Parents whose children meet these criteria were informed about the study in detail, and informed consent was obtained from both the youth and their parents who agreed to take part in the study.

All subjects and their parents were interviewed by the clinicians, individually. Information like sociodemographic characteristics, time spent on screen, screen use habits like gaming, video watching, chatting, number of devices owned by youth and accessible to youth, were obtained. For subjects when parent and youth information on screen time was not consistent, parent’s information was preferred. The capability of parents to impose limits on youth’s screen usage, and time spent on screen by parents was also examined.

After the initial evaluation, parents of the group ADHD + LD were given the Mathematical and Literacy Assessment Scale, a scale developed in Turkey in which, higher scores correlate with the severity of learning disorders [20].

The youth were given Young Internet Addiction Scale [21, 22], Internet Gaming Disorder Scale [23], Smartphone Addiction Scale [24], and Social Media Addiction Scale [25, 26]. These internet-related scales were selected to assess different domains of technology use behaviors. While the Young Internet Addiction Scale provides a general measure of problematic internet use, the other scales target more specific forms of digital engagement, such as online gaming, smartphone use, and social media activity.

### Statistical analysis

The collected data were analyzed using SPSS v22.0. Data were examined for outliers and missing values. The Kolmogorov–Smirnov test was used to assess normality. Groups were compared using the chi-square test for categorical variables, and Fisher’s exact test was applied when necessary. For continuous variables, the independent samples t-test was used or the Mann–Whitney U test was employed if required. In order to investigate the relationship between the variables, Pearson and Spearman correlation analyses were performed. An alpha level of 0.05 was set as the threshold for statistical significance in the analyses. To control for the potential risk of Type I error due to multiple comparisons, a False Discovery Rate adjustment was applied using the Benjamini–Hochberg procedure, and adjusted p values were reported where relevant.

## Results

Average age for the ADHD + LD group and the ADHD-only group were 11.82 ± 2.44 and 11.96 ± 1.96, respectively (*p* > 0.05). Both groups consisted of 60 males and 26 females (*p* > 0.05). There were no statistically significant difference between the groups in terms of age, gender distribution, financial status of the families, and parental screen time control capabilities (*p* > 0.05). The ADHD + LD group had higher rates of grade failure history (*p* < 0.001, X^2^_(1)_ = 14.69) (Table 1).

**Table 1:** Comparison of participants’ sociodemographic data

		ADHD + LD (*n* = 86)	ADHD (*n* = 86)	df	Effect size	*p*	Adjusted *p*
Age^a^, Mean ± SD		11.82 ± 2.44	11.96 ± 1.96	162.351	Cohen’s d (95% CI) = 0.062 (-0.237, 0.361)	0.683	0.872
Gender^b^, M: F		60:26	60:26	1	Cramer’s V < 0.001	1.000	1.000
Family Income ^b^, TL / mo, n (%)	< 18.000	18 (20.9)	11 (12.8)	3	Cramer’s V = 0.177	0.220	0.440
	18.000–30.000	29 (33.7)	27 (31.4)				
	30.000–50.000	25 (29.1)	24(27.9)				
	> 50.000	14 (16.3)	24 (27.9)				
History of Grade Retention ^b^, n (%)		16 (18.6)	1 (1.2)	1	Cramer’s V = 0.292	< 0.001	< 0.001
Self-Reported Academic Performance^b^, n (%)	Good	41 (47.7)	58 (67.4)	2	Cramer’s V = 0.202	0.030	0.090
	Average	37 (43.0)	24 (27.9)				
	Poor	8 (9.3)	4 (4.7)				
Subject to Parental Limits ^b^, n (%)		21 (24.4)	23 (26.7)	1	Cramer’s V = 0.027	0.727	0.872

*Abbreviations*: SD, standard deviation; df, degrees of freedom

^a^ Tested by independent samples t test

^b^ Tested by Chi Square test

Both groups had similar results in terms of screen time (*p* > 0.05). ADHD + LD group showed less variety in terms of device usage (*p* = 0.004, t_(170)_= -3.30), with less usage of mobile phones (*p* = 0.038, X^2^_(1)_ = 4.79) and personal computers (PC) (*p* = 0.016, X^2^_(1)_ = 7.07). Screen usage purposes were also less varied in the ADHD + LD group, with less usage for chatting (*p* = 0.026, X^2^_(1)_ = 6.37), listening to music (*p* = 0.004, X^2^_(1)_ = 10.43), watching movies (*p* = 0.026, X^2^_(1)_ = 6.00) and videos (*p* = 0.041, X^2^_(1)_ = 4.76), and education-based activities (*p* < 0.001, X^2^_(1)_ = 19.16) (Tables 2 and 3).

**Table 2:** Comparison of the variety of Screen-Based device usage among participants

	ADHD + LD (*n* = 86)	ADHD (*n* = 86)	df	Effect Size	*p*	Adjusted *p*
Screen Time per day ^a^, h Mean ± SD	4.27 ± 2.49	3.99 ± 2.57	170	Cohen’s d (95% CI) = 0.110 (-0.189-0.409)	0.474	0.569
Total Number of Devices Used (Phone, Tablet, Computer, Game Console)^a^ Mean ± SD	1.92 ± 0.88	2.35 ± 0.82	170	Cohen’s d (95% CI) = 0.504 (0.199–0.807)	0.001	0.004
Phone Use^b^ n (%)	72 (83.7)	81 (94.2)	1	Cramer’s V = 0.167	0.029	0.038
Computer Use^b^ n (%)	44 (51.2)	61 (70.9)	1	Cramer’s V = 0.203	0.008	0.016
Tablet Use^b^ n (%)	35 (40.7)	44 (51.2)	1	Cramer’s V = 0.105	0.168	0.192
Game Console Use^b^ n (%)	14 (16.3)	16 (18.6)	1	Cramer’s V = 0.031	0.688	0.688

*Abbreviations*: SD, standard deviation; df, degrees of freedom

^a^ Tested by independent samples t test

^b^ Tested by Chi Square test

**Table 3:** Comparison of participants’ purposes for screen use

	ADHD + LD (*n* = 86) n (%)	ADHD (*n* = 86) n (%)	df	Cramer’s V	*p*	Adjusted *p*
Social Media	52 (60.5)	45 (52.3)	1	0.082	0.282	0.282
Messaging	46 (53.5)	62 (72.1)	1	0.192	0.012	0.026
Video watching	77 (89.5)	84 (97.7)	1	0.166	0.029	0.041
Music listening	38 (44.2)	59 (68.6)	1	0.246	0.001	0.004
Movie watching	37 (43.0)	53 (61.6)	1	0.186	0.015	0.026
Educative Activities	33 (38.8)	62 (72.1)	1	0.335	< 0.001	< 0.001
Gaming	81 (94.2)	76 (88.4)	1	0.103	0.177	0.207

*Abbreviations*: SD, standard deviation; df, degrees of freedom

All variables were compared using the chi-square test

Game subtype preferences revealed that the ADHD + LD group tends to prefer playing Multiplayer Online Battle Arena (MOBA) (X^2^_(1)_ = 14.10, *p* = 0.002), Real-Time Strategy (RTS) (X^2^_(1)_ = 13.14, *p* = 0.004), and Puzzle & Platform (X^2^_(1)_ = 10.58, *p* = 0.004) games less (Table 4).

**Table 4:** Comparison of game subgroups played by participants engaging in Screen-Based gaming activities

	ADHD + LD (*n* = 81), n (%)	ADHD (*n* = 76) n (%)	X^2^	df	Cramer’s V	*p*	Adjusted *p*
Battle Royale	33 (40.7)	24 (31.6)	1.42	1	0.095	0.233	0.325
FPS	22 (27.2)	31 (40.8)	3.26	1	0.144	0.071	0.188
MOBA	29 (35.8)	50 (65.8)	14.10	1	0.300	< 0.001	0.002
MMORPG	0 (0.0)	2 (2.6)	FET^a^		0.117	0.233	0.325
Survival	42 (51.9)	43 (56.6)	0.35	1	0.047	0.552	0.552
Role-Playing	35 (43.2)	43 (56.6)	2.80	1	0.134	0.094	0.188
RTS	2 (2.5)	16 (21.1)	13.14	1	0.290	0.001	0.004
Puzzle &Platform	27 (33.3)	45 (59.2)	10.58	1	0.260	0.001	0.004
Online Card-Board	2 (2.5)	4 (5.3)	FET^a^		0.073	0.431	0.470
Simulation	33 (40.7)	38 (50.0)	1.36	1	0.093	0.244	0.325
Sports	14 (17.3)	18 (23.7)	0.99	1	0.079	0.320	0.384
Racing	23 (28.4)	13 (17.1)	2.83	1	0.134	0.093	0.188

*Abbreviations*: FPS, First Person Shooter; MOBA, Multiplayer Online Battle Arena; RTS, Real Time Strategy; MMORPG, Massively Multiplayer Online Role-Playing Game; FET, Fischer Exact test; SD, standard deviation; df, degrees of freedom

^a^ FET indicates that Fischer Exact Test was performed, Chi-Square test was performed for other comparisons

No significant difference was found in Young Internet Addiction Scale, Internet Gaming Disorder Scale, Smartphone Addiction Scale and Social Media Addiction Scale scores between groups (*p* > 0.05). Mathematics and Literacy Assessment Scale scores showed no correlation with other scale scores, or screen time per day (*p* > 0.05).

Regrouping the entire sample based on parents’ ability to set limits on screen time revealed that the group with imposed limits was younger (M = 10.88, SD = 1.96, *n* = 44 vs. M = 12.24, SD = 2.19, *n* = 128, t_(170)_ = 3.63, *p* < 0.001) and had higher self-reported academic performance scores (X^2^_(2)_ = 9.47, *p* = 0.021). They also spent less time on screens (Median = 0.75 h, IQR = 0.33–2.00 vs. Median = 4.71 h, IQR = 4.00–6.33, Z= -8.41, *p* < 0.001). The variety of device usage between the groups was similar; however, regarding screen usage purposes, the group with restrictions showed less interest in social media (X^2^_(1)_ = 17.33, *p* < 0.001) and chatting (X^2^_(1)_ = 14.76, *p* < 0.001). The groups did not differ in terms of gender distribution, family income, or grade retention history (*p* > 0.05).

An evaluation conducted on the entire sample, independent of groups, revealed positive-weak correlations between the number of screen-based devices owned by youth and screen time per day (*p* = 0.007, *r* = 0.204), and duration of device ownership and screen time per day (*p* < 0.001, *r* = 0.282). No significant correlation was found between number of screen-based devices accessible to youth and screen time per day (*p* > 0.05). Additionally, no significant correlation was found between duration of methylphenidate treatment and screen time per day (*p* > 0.05) (Table 5).

**Table 5:** Correlations between daily screen time, methylphenidate treatment duration, and device usage variables

	Duration of methylphenidate Treatment ^a^	Number of Accessible Devices ^a^	Duration of Device Ownership, y^a^	Number of Owned Devices^b^
Screen Time Per day, h	*r* = 0.116, *p* = 0.129	*r* = 0.142, *p* = 0.064	*r* = 0.282, *p* < 0.001	*r* = 0.204, *p* = 0.007

^a^ Pearson Correlation Analysis was performed

^b^.Spearman Correlation Analysis was performed

**Fig. 1:**
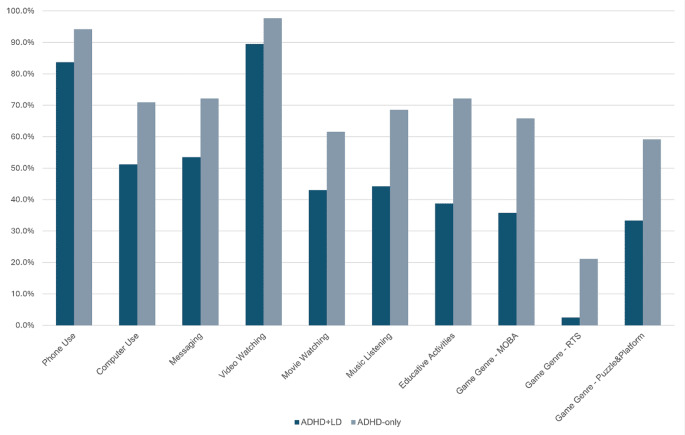
Bar plot illustrating the proportion of adolescents in the ADHD-only and ADHD + LD groups who reported engagement in specific screen-based behaviors. The figure summarizes only those activities for which statistically significant group differences were found in the statistical analyses. The total number of devices used, although statistically different between groups, was not included due to scale-related visualization constraints

A consolidated summary of screen-based activities with significant differences between the ADHD-only and ADHD + LD groups is presented in Fig. 1. Although the total number of devices used differed significantly between the groups, this parameter was not included in the main bar plot due to scale-related visualization constraints. Given its smaller range of values compared to proportional activity data, it was presented in the main text and tables instead.

## Discussion

This study explores the potential effects of LD comorbidity on screen-based device usage patterns. While there are similarities between the groups in some assessment categories, we believe that several differences warrant further examination. Before delving into the discussion of screen-based behaviors, it is important to address a notable issue regarding self-reported academic performance. Although the ADHD + LD group initially appeared to report lower academic achievement, this difference was not statistically significant when evaluated using adjusted p-values (*p* = 0.09). While it would be expected for the ADHD + LD group to show lower academic performance, this anticipated difference may have been influenced by several factors—such as defensive response tendencies, the inherent limitations of self-reported data, or discrepancies between perceived and actual academic functioning. As comparing academic performance between groups was not a primary focus of this study and was assessed only as a supplementary variable, we did not collect objective academic performance measures in a way that would allow direct comparison between groups. Nonetheless, it is worth noting that the two groups did differ significantly in terms of grade retention history, which may serve as a relatively more objective indicator of academic challenges and appears consistent with expectations regarding the ADHD + LD group. Therefore, we were unable to verify this finding through independent data, and it should be interpreted with caution.

Our findings indicate that, for the ADHD + LD group, while there is no difference in daily screen time, there are differences in the usage patterns, compared to the ADHD-only group.

In terms of device usage, the ADHD + LD group was less likely to use PCs. Considering that groups were sociodemographically and financially similar, this result is most likely explained with youth preferences, rather than parents’ financial providing capabilities. Computers are more complex devices to learn and use compared to their alternatives, partly due to their more intricate user interfaces, and this is one possible reason why youth with LD may be less likely to use PCs [27, 28].

The ADHD-only group was also more inclined to use mobile phones and showed higher rates of usage for chatting, listening to music, watching movies/videos and engaging in educational activities.

It is undeniable that texting and chatting are the crucial parts of our daily lives. Today, there are many types of tablets on the market, with accompanying SIM cards and mobile internet connectivity, offering functions quite similar to those of mobile phones. However, mobile phones are more portable for daily use compared to the tablets, and the advantage on accessibility may explain the preference over tablets. From this perspective, it is understandable that the ADHD-only group, with higher texting rates, may tend to prefer mobile phones compared to the ADHD + LD group.

There are numerous digital applications and programs designed to assist youth with LD worldwide. Although widely used abroad, their use is limited in our country. On the other hand, there are numerous channels on social media platforms like Youtube and Instagram, creating contents aimed to help youth with formal education curriculum. However, considering that the target audience of those channels are healthy youth, the contents of those channels may be difficult to follow for youth with LD. This could explain why the technology use for educational purposes, which is expected higher in the ADHD + LD group, was found more frequent in the ADHD-only group. To conclude, it can be deduced that the ADHD + LD group tends to have fewer varied activities involving screen usage, while the ADHD-only group demonstrates more sophisticated and task-oriented screen use for various purposes.

Gaming and social media usage rates were similar between two groups. However, our study found those in the ADHD + LD are less prone to play MOBA, RTS and Puzzle & Platform game subgroups.

RTS is a term used to describe game types, where the goal is to dominate the opponent through resource management [29]. Age of Empires, Starcraft and Warcraft are well-known examples of the genre.

MOBA, which can also be defined as an RTS subgenre, refers to the games where each player controls a single character on a corridor with specific abilities, using resources to enhance their character’s certain abilities based on their teammates’ and opponents’ characteristics [30]. League of Legends, Defense of the Ancients, Heroes of the Storm, Brawl Stars are games that can be classified under this group.

Puzzle & Platform games, as the name suggests, are games where obstacles and puzzles must be overcome in order to progress. The range of this game group is broader compared to the aforementioned two subgroups, as both a 2D linear mobile game and a complex PC game like Portal are considered members of this group. Moreover, although not originally classified as puzzle games, horror/survival games like Amnesia and Outlast requires significant puzzle-solving skills, where next step is not explained and the players are pushed to find solutions under time pressure, to progress to the next level.

Evaluating these game subtypes within the context of the aforementioned knowledge, those game genres may require certain skills related to executive functions to succeed [31]. For example, in League of Legends, when the opponents have retreated to their center, in their absence, the player has limited time window to contest resources controlled by the opponents and gain an advantage. However, while doing that, the players need to calculate how long it will take for the opponents to return, monitor other lanes to ensure that all other opponents are in their lanes, as they may leave from time to time and gank (execute a coordinated attack to overwhelm the player) them. Moreover, the players need to stay alert all the time to actively track opponents’ skills and abilities’ cooldowns to decide when to attack, or when to retreat. When engaging the opponent, or when the players are being engaged, they need to react fast, counteract if possible, and shift focus to different targets for better outcomes.

There are studies suggesting that comorbidity with LD has a negative impact on executive functions in youth with ADHD; they have been found to exhibit weaker working memory and task-switching abilities compared to youth without LD comorbidity [32]. Additionally, youth with LD also demonstrate lower levels of coordination skills [33]. Moreover, gaming, especially RTS and MOBA subtypes, require teamplay and constant need of communication. Although voice chat is a more efficient option, the majority of communication in games is still occurs through text-based messsaging. That requires certain level of typing and reading skills which can be tough for youth with LD, especially for dyslexia subgroup. In light of this information, it can be speculated that youth with LD may find those game subtypes more challenging to play, and prefer these subgenres less, compared to their peers.

The results of our research also revealed that although there is no correlation between screen time per day and number of screen-based devices accessible to youth, there is a positive correlation between screen time per day and number of screen-based devices owned by youth. To the extent of our database searches, we did not find any other research examining the duration of screen-based device ownership, number of screen-based devices owned by youth and number of screen-based devices accessible to youth and their correlation between screen time per day. To our knowledge, our research is the first study in the literature evaluating the relationship between these three parameters and screen time per day.

The finding that, number of devices owned by youth shows positive correlation while number of devices accessible to youth showing no correlation at all can be explained by the indirect limiting effect on the availability of devices for youth’s use. Devices owned by parents or shared with siblings are not always available for youth. Conversely, when the devices are not occupied, the youth may be busy with daily tasks and other activities, thus limiting time spent on screen. As expected, when the youth have their own devices, they have more opportunities to use it for the same reasons; as the number of devices owned by youth increases, so does screen time, since they have more options to turn to.

On this matter, limiting screen-based device ownership may be more challenging in practice than it might seem. Youth require PCs or tablets to use for their homework. It is also understandable that parents’ concern for their children’s needs and well-being, and being able to contact them in times of need. Therefore, many parents buy their children mobile phones and although it is bought with good intentions, the unintended consequences cause the problem. Under these circumstances, especially for smaller children, who are less likely to regulate themselves on time spent on screen, smartwatches with SIM card functionality might offer a better alternative, which are similar to mobile phones in terms of communication capabilities, and less likely to be overused by youth due to limited features.

Our results showed a correlation between the increase in the duration of ownership of a screen-based device and screen time per day. Neuroimaging studies show certain alterations in the reward circuits of the brain in the individuals with IGD, just like other addictions [19]. Cognitive Behavioral Approach also theorizes that gaming behavior, as a source of pleasure, creates a self-reinforcing cycle [34]. Combining these insights with the previously discussed negative effects of owning a screen-based device, such a result is not surprising.

IGD is a diagnosis with a well-known comorbidity of ADHD. It’s thought that impulsivity and self-regulation difficulties in ADHD play a role in IGD etiopathogenesis. This theory led to treating IGD with ADHD pharmacotherapy, which led to beneficial results [35]. Another study demonstrated improved outcomes in treating IGD patients with Methylphenidate over a 3- month follow-up period, compared to the control group [36]. Our results indicate no correlation between methylphenidate treatment duration and screen time per day. Since the primary aim of our study to examine the possible effects of LD comorbidity on youth with PTU, the participants were recruited from patients who had been receiving methylphenidate treatment with at least 6 months, to eliminate potential confounding factors. Therefore, it can be deduced that the majority of the benefits of methylphenidate treatment on IGD comorbidity emerge within 6 months of the treatment, while the following period primarily contributes on maintaining the achieved outcomes.

Our results also show that youth who have imposed limits on screen time tend to be younger, spend less time on screens, and engage less frequently in social media and chatting. Younger children, who are more likely to comply with parental authority, are easier for parents to manage in terms of limiting screen time, as expected. It’s also understandable that younger children prefer to spend less time on these activities, because they are more adolescent and adult-oriented activities. We might speculate that, these younger children could also be interested in these activities; however, they prefer to spend their limited and valuable screen time playing games; which is expected to be the most pleasurable activity for them.

While the overall levels of problematic technology use did not differ significantly between groups, the differences observed in usage patterns may still carry clinical relevance. Rather than focusing solely on the duration of screen use, clinicians might benefit from considering what purposes screen time serves for each individual. For instance, if a child’s engagement with technology is partly shaped by the relative difficulty they experience with academic tasks, rather than being driven by impulsivity, it may point to different underlying needs and require distinct clinical considerations. Furthermore, exploring what children do during screen time, such as the type of content they engage with or the devices they prefer, may offer indirect clues about potential comorbidities. A child who consistently avoids cognitively demanding games or prefers simpler interfaces, such as tablets instead of computers, might be exhibiting signs related to underlying learning difficulties. Thus, assessing usage patterns in more detail could support a more nuanced understanding of the child’s functional profile.

### Limitations

Our study is among the first to investigate the relationship between PTU and LD in ADHD-diagnosed children. However, it is not without limitations. Being a single-center study represents a limitation regarding the generalizability of our findings. Additionally, the relatively small sample size may have limited the statistical power of our analyses and prevented more detailed subgroup evaluations—particularly in relation to participant-level characteristics such as age and gender, which could offer further insights given that technology use preferences may vary across these dimensions.

Furthermore, as our study was cross-sectional in design, causal inferences could not be established. Rehabilitative educational approaches for LD may have an effect on the relationship between PTU and LD. Nevertheless, due to the variability in the types and durations of educational modules provided by different institutions, standardization could not be achieved, and this factor was excluded from the study.

We did not have the opportunity to evaluate thoroughly the hardware specifications of the devices used by youth. Considering that high-performance devices may offer more options, particularly in the gaming domain, we believe, this may be also a potential limiting factor of our study.

## Data Availability

The datasets generated and analyzed during the current study are available from the corresponding author on reasonable request.
